# Human–Robot Interaction in Agriculture: A Systematic Review

**DOI:** 10.3390/s23156776

**Published:** 2023-07-28

**Authors:** Lefteris Benos, Vasileios Moysiadis, Dimitrios Kateris, Aristotelis C. Tagarakis, Patrizia Busato, Simon Pearson, Dionysis Bochtis

**Affiliations:** 1 Institute for Bio-Economy and Agri-Technology (IBO), Centre of Research and Technology-Hellas (CERTH), Charilaou-Thermi Rd, 57001 Thessaloniki, Greece; e.benos@certh.gr (L.B.); v.moysiadis@farm-b.com (V.M.); d.kateris@certh.gr (D.K.); a.tagarakis@certh.gr (A.C.T.); 2 Department of Computer Science and Telecommunications, University of Thessaly, 35131 Lamia, Greece; 3 FarmB Digital Agriculture S.A., 17th November 79, 55534 Thessaloniki, Greece; 4 Interuniversity Department of Regional and Urban Studies and Planning (DIST), Polytechnic of Turin, Viale Mattioli 39, 10125 Torino, Italy; patrizia.busato@unito.it; 5 Lincoln Institute for Agri-Food Technology (LIAT), University of Lincoln, Lincoln LN6 7TS, UK; spearson@lincoln.ac.uk

**Keywords:** human–robot synergy, collaborative robotics, communication frameworks, human-centered automation, agriculture 4.0

## Abstract

In the pursuit of optimizing the efficiency, flexibility, and adaptability of agricultural practices, human–robot interaction (HRI) has emerged in agriculture. Enabled by the ongoing advancement in information and communication technologies, this approach aspires to overcome the challenges originating from the inherent complex agricultural environments. Τhis paper systematically reviews the scholarly literature to capture the current progress and trends in this promising field as well as identify future research directions. It can be inferred that there is a growing interest in this field, which relies on combining perspectives from several disciplines to obtain a holistic understanding. The subject of the selected papers is mainly synergistic target detection, while simulation was the main methodology. Furthermore, melons, grapes, and strawberries were the crops with the highest interest for HRI applications. Finally, collaboration and cooperation were the most preferred interaction modes, with various levels of automation being examined. On all occasions, the synergy of humans and robots demonstrated the best results in terms of system performance, physical workload of workers, and time needed to execute the performed tasks. However, despite the associated progress, there is still a long way to go towards establishing viable, functional, and safe human–robot interactive systems.

## 1. Introduction

### 1.1. Background

Robots and autonomous systems exploit their capability to sense, scrutinize, analyze, and interact with the physical environment without or with minimal human intervention [1]. Focusing on the agricultural sector, the advent of robotic systems is envisioned to contribute to ending hunger and malnutrition in a sustainable manner by conserving and restoring ecosystems and natural resources [2,3,4]. Robots are considered as an integral element of Agriculture 4.0, which comes as an evolution of precision agriculture, enabling farmers to utilize the minimum required quantities for specific areas. Agri-robots belong to a broad family of Information and Communications Technologies (ICT), also including, indicatively, wireless sensor networks, farm management information systems, cloud computing, big data, and artificial intelligence, that are prerequisites for the fourth agricultural revolution [5,6].

Taking advantage of the advancement of ICT, along with the reduction in the corresponding costs, because of mass production, robots are being more and more implemented in agriculture [7]. Robotic systems can increase agricultural productivity, as they optimize the efficiency of the implemented agricultural practices. In addition, robots have the potential to take humans out of hazardous locations and address labor shortages of seasonal workers [8]. Remarkably, the recent coronavirus pandemic has resulted in a spike in investment in agri-robotics as a means of filling labor shortages [9,10]. Indicative examples of agri-robot tasks are also sowing and planting, spraying, weeding, land preparation, insect and disease detection, plant monitoring, and phenotyping [11,12,13]. Moreover, multi-purposed robots have been developed, thus adding intricacy to both software and hardware and leading to increased costs [14].

In general, robots are able to carry out repetitive and predetermined assignments in stable environments and are closely related to tasks belonging in the so-called “three D’s”, namely dull, dirty, and dangerous tasks [15]. Unlike industrial settings, which contain a stable environment with well-structured objects, agriculture is characterized by uncertainty, heterogeneity, and unpredictable situations. Therefore, advanced technologies must cope with highly complicated environments, variable physical conditions, and live produce, which necessitates gentle and precise manipulations. More specifically, illumination, terrain, and other atmospheric conditions are ill defined, while there is a high variability in crop color, shape, and position that cannot be determined a priori [16]. These features render the replacement of humans by autonomous robots in agriculture very challenging [17].

### 1.2. The General Context of Human–Robot Interaction in Agriculture 

#### 1.2.1. Human–Robot Interaction Definition

With the intention of addressing the challenges provoked by complex agricultural environments, the synergy of humans and robots has been proposed. Human–robot interaction (HRI) constitutes a multidisciplinary research field dealing with investigating, designing, and evaluating these collaborative systems. It combines artificial intelligence, robotics, ergonomics, engineering, computer science, and social science to endow robots and humans with all the required competencies for proper interaction. In particular, HRI refers to the process whereby humans act as a team with robots to achieve a goal and comes from the confluence of information exchange, autonomy, and optimal task shaping [18]. HRI integrates the distinctive cognitive human skills of dexterity, perception, judging, and decision making with those assets of robots concerning repeatable accuracy and strength. The developed robot cognitive capabilities are a result of the integration of several sensors such as laser scanners, radio-frequency identification (RFID), cameras, and actuators. This innovative combination enables versatile use, robustness, flexibility, and adaptability under a constantly evolving workflow [19]. HRI can be accomplished via proximal or remote interaction. The ultimate objective of HRI is to free humans from dangerous and routine tasks. For instance, in the case of pesticide spraying, there can be an operator directing or supervising the task from a safe distance and away from harmful chemicals with the use of a properly designed user interface. These semi-autonomous systems have demonstrated remarkable results, outperforming fully autonomous robots [20]. In short, human–robot synergy can provide many advantages, including flexibility when it comes to system reconfiguration, reduction in the required working area, increasing productivity, improvement of the quality of services, rabid capital depreciation, and the creation of highly skilled jobs [21].

#### 1.2.2. Main Design Concepts 

One of the most challenging issues in HRI is the design of these synergistic systems, owing to the wide range of different working conditions and levels of interaction that may be faced. Human operators can be easily accused of being responsible for “human error” when they fail to notice an off-nominal instance. Nevertheless, insufficient design of the system and the associated interactions can lead to less-than-optimal compensatory reaction of the humans [22]. Every betterment of HRI is based predominantly on two principles: the autonomy level of the robot and the closeness of human and robot during their interaction. The level of autonomy that these interactive systems can achieve relies on strategies that enable HRI in such an adaptable way that humans can intercede when it is required. In broad terms, the design should not limit the visual perspectives and mobility of humans or include inconvenient software. Also, robots should be programmed with cognitive skills to interact in an accurate and fluid manner, thus guaranteeing the dynamic autonomy of the system. In addition, different situations should be investigated in relation to proximity, such as following, passing, avoiding, and touching. The design of human–robot interactive systems should also consider the human-to-robot ratio along with the specific roles of the former (programmer, bystander, operator, supervisor, and information consumer). Design concepts also pay attention to adaption, task shaping, and the working time during which humans and robots coexist in the same workspace, while every objective has to match with the next one [23]. 

#### 1.2.3. Communication Frameworks

Interaction, by definition, calls for the development of communication frameworks, which aspire to simplify the knowledge sharing between robots, or machines in general, and humans. In essence, more natural ways of communicating need to be investigated, such as body language and vocal communication. The former term encompasses facial expressions, body postures, and hand gestures, whereas the latter is limited by the noisiness of agricultural environments and the dissimilar ways that someone may pronounce a command. Out of these communication channels, hand gesture recognition, either through acquisition of data from vision sensors or specially designed gloves, has attracted the interest of the scientific literature [24,25]. Furthermore, surface electromyography sensors have been used for recording the electric potential of muscles [26], while hybrid methodologies have also been examined [27]. In brief, the main shortcomings of the above approaches are as follows: (a) vision sensors run into problems whenever changes take place with many people, complex backgrounds, and illumination changes [28]; (b) gloves usually limit natural movements [29]; and (c) electromyography sensors generate massive and noisy datasets [30]. Although the literature on the development of non-verbal communication tools in agriculture is still scarce, some efforts have been presented with encouraging results [24,31]. Finally, face recognition has not yet been widely used in agricultural environments due to the above-mentioned problems associated with vision sensors as well as restrictions imposed by privacy policies [32]. 

#### 1.2.4. Safety and Human Factors

The primary concern concerning these fenceless synergistic systems is to ensure safety and health of humans and disclose all the risk factors that may harm them [33]. Occupational health in centered upon improving the shared workspace to help workers avoiding risky postures that can potentially cause injuries (physical ergonomics). In addition aspects, like mental workload and work stress are taken into consideration (cognitive ergonomics) [34]. On the other hand, occupational safety includes accident control measures. Overall, occupational health and safety can impact the efficiency of the system, response time, quality of work, and collaborative performance. Accordingly, an optimal synergistic human–robot system should be designed from the perspective of mental welfare, psychological comfort, and occupational health and safety. These aspects are related to perceived safety. The key elements that determine perceived safety are considered to be predictability, sense of control, experience, familiarity, transparency, comfort, and trust [35,36]. As a final note, only authorized and qualified workers must work together with a robot, while attention is paid to the establishment and evaluation of safety protocols and risks. The latter must be thoroughly investigated in the design phase, as unweighted factors, including uncertainty in interpreting and possible failures of human or robots, may take place during HRI. 

#### 1.2.5. Human–Robot Interaction Evaluation and Metrics

The design of synergistic systems necessitates the consideration of the implications of automation on the performance of both robot and human as a means of optimizing the overall benefits for the system. As a result, a significant feature of the design of collaborative tasks is the appraisal of their performance, fluency, effectiveness, and adaptability through adequate metrics allowing for reproducible evaluations. Several studies are concerned with metrics for HRI [37,38,39,40]. Indicatively, Vásconez et al. [23] summarized the main metrics that have been studied for evaluating the synergistic systems [41,42] and grouped them into six categories in relation to their usage, namely (a) mission effectiveness (e.g., performance of the mission); (b) human behavior efficiency (e.g., decision making and problem recognition); (c) human cognitive indicators (e.g., situation awareness, trust in robotic systems, and situation awareness); (d) human physiological indicators (comfort and fatigue); (e) robot behavior efficiency (e.g., autonomy level, human awareness, and learnability); and (f) collaborative metrics (e.g., collaborative problem recognition and action implementation efficiency, team situation awareness, and social patterns and roles). As stated in [40], the metrics do not entirely measure the impact of the autonomy level on interaction, since they normally focus on the observation of either humans or robots and not on their capabilities, therefore introducing error in analysis. As a general remark, it is very difficult to evaluate such kinds of systems in a broad and objective assessment. Moreover, the lack of efficient human-in-the-loop assessment has made it problematic to conclude whether such adaptation could bring about satisfying HRI [43]. Finally, the majority of relevant studies are limited to how the robotic system affects the human factors without, however, focusing on the opposite; how human factors impact the system [22].

#### 1.2.6. Aim and Structure of the Paper

This paper provides a systematic review investigating the state of the art in HRI and the main challenges that must be addressed, focusing solely on the field of agriculture. The research is conducted through the lenses of different aspects by screening the relevant scholarly literature based on the PRISMA guidelines [44]. The remainder of the present paper is structured as follows. Section 2 describes the implemented methodology for the bibliographic survey, how the methodological quality of the selected studies and level of automation were evaluated, and the classification framework that was used. The results are analyzed in Section 3, also including the list of the selected papers and related statistics. Finally, Section 4 contains the main conclusions of the present review study, along with a discussion from a broader perspective to identify future research directions.

## 2. Materials and Methods

### 2.1. Critical Steps in Performing the Systematic Review

A systematic review is considered a rigorous approach to literature review that involves identifying, synthesizing, and evaluating all the available scientific evidence, both qualitative and quantitative. They are used to produce a robust, empirically derived response to a research question related to a specific topic. By adhering to systematic review principles, they offer distinct advantages over traditional literature reviews. These advantages include enhanced review quality through increased transparency, improved objectivity, and mitigation of researcher bias. Additionally, systematic reviews encourage researchers to critically assess the quality of evidence, thus strengthening the overall review process. While systematic reviews provide comprehensive and unbiased insights, their validity can be influenced by factors such as variations in evidence availability and quality, potential study selection biases, resource limitations, and challenges in addressing complex research inquiries. Nevertheless, systematic reviews remain invaluable tools for evidence synthesis, enabling informed decision making, statistical robustness, and identification of significant patterns and trends. It is important, however, to interpret their findings cautiously within the appropriate contextual framework.

In the present systematic review, seven steps were used in a manner similar to the relevant literature [45,46]:

(1) Formulation of a primary research question: “What is the state of the art and what are future perspectives in HRI in agriculture?”
(2) Development of a research protocol: The methodology followed for screening the relevant literature and data extraction and analysis was included in a written document. This was accepted by all the authors of this study, prior to the start of the literature search, to minimize bias.
(3) Literature search: The methodology for selecting the relevant studies is described in Section 2.2 along with the implemented electronic databases, inclusive criteria, and review stages based on the PRISMA guidelines [44].
(4) Data extraction: Specific items, regarding references (including journal, title, and authors), objective, method, crop type, interaction modes, automation levels, and key outcomes, were gathered in an online shared spreadsheet.
(5) Quality appraisal of the selected studies: Although quality remains a challenging concept to define, the present study used the tool developed by Hoy et al. [47] (described in Section 2.3), which comprises specific internal and external validity criteria.
(6) Data analysis and results: The first step in this procedure included a simple descriptive assessment of each study, presented in tabular form, followed by a statistical analysis.
(7) Interpretation of results: Conclusions were drawn based on the available scientific evidence, while areas were identified to focus on for future research.

### 2.2. Literature Search

**Figure 1:**
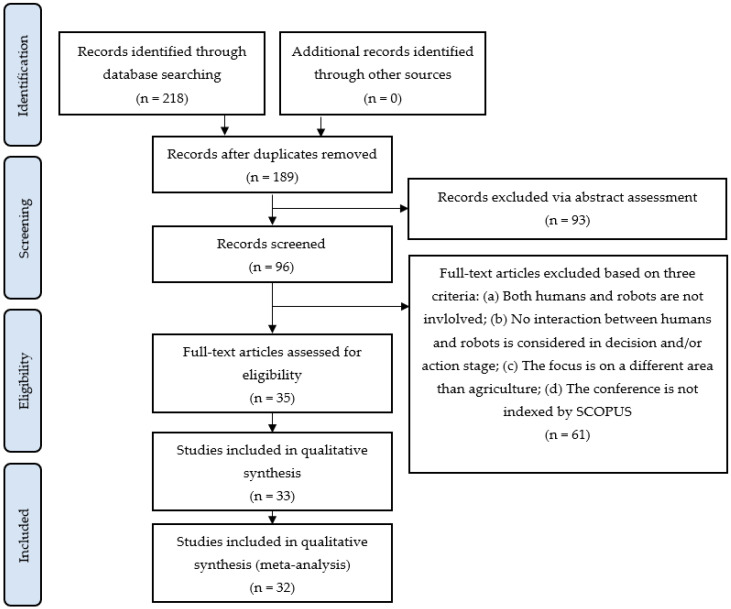
Flow diagram regarding the present systematic review process for selecting the relevant studies.

The search engines of Google Scholar, ScienceDirect, Scopus, ΙΕΕΕ Xplorer, and MDPI were used for the purpose of seeking publications associated with HRI in agriculture. To that end, Boolean keyword combinations of “human-robot interaction/collaboration/synergy” and “agriculture” were used. Subsequently, the references of each article were scanned with the intention of finding studies that had not been noticed during the initial search. This process was reproduced until there were no more relevant publications. The ultimate search was performed on 15 December 2022. The titles and the abstracts of the resulting papers were then reviewed. As a next step, the full text of the relevant studies was carefully read to ascertain their appropriateness. For the selection of the final scientific literature to be considered, the following criteria should be met: (a) both humans and robots are involved; (b) HRI is considered in the decision and/or action stage; (c) I application domain is agriculture; (d) conference papers are also included, provided that the conference is indexed by SCOPUS. Non-English studies, Master theses, and doctoral dissertations were not included in the research. A final consensus meeting of the co-authors was held to discuss the content and adequacy of the selected papers based on the above criteria and resolve any difference of opinion. A flowchart summarizing the implemented methodology of the present systematic review is depicted in Figure 1, based on the PRISMA guidelines [44] for transparently reporting how the relevant literature was selected. The bibliographic survey on HRI in agriculture resulted in 32 relevant studies that fulfill the imposed inclusion criteria, of which 21 are journal papers and 11 are conference papers.

### 2.3. Methodological Quality Assessment

Assessing the risk of bias of the methodology applied in the selected investigations is very crucial for interpreting literature reviews so as not to underestimate or overestimate their results. In this review study, the risk of bias tool developed by Hoy et al. [47] was considered. This tool is made of 4 and 6 items with reference to external and internal validity criteria, respectively, accompanied by a summary item corresponding to the overall assessment of the quality of the methodology. The first ten items are yes/no questions oriented toward detecting potential bias in measurement methods. If no insufficient information exists, the corresponding answer is “No” [47]. For studies that do not involve participants, such as those developing mathematical models, using simulations, or dealing with design principles, some items may be filled in with “C”. This letter stands for “Can’t say”, similar to [48,49]. These items were not taken into consideration in the final summary item. All the authors of this paper independently took part in the reviewing process by answering all the questions to assess the risk of bias of the methodology for each study. A consensus meeting was held to compare the results and find a commonly accepted final answer. Additional criteria were applied pertaining to “C” cases, such as reliable measurement method and appropriate methodology validation.

As far as the eleventh summary item is concerned, which represents the overall methodological quality, it was rated as follows:

- High (++), indicating low risk of bias;
- Acceptable (+), indicating moderate risk of bias;
- Low (−), indicating high risk of bias.

In practice, depending on the number of “Yes” answers in the first 10 items of the tool of Hoy et al. [47], each paper was scored in the range 0–100% (each “Yes” answer has a 10% contribution to the final score). Similar to [49], 75% was considered as the lower limit, beyond which high (++) overall quality of the methodology was established. Moreover, scores between 50% and the above limit were rated as acceptable, while those below 50% represent studies with relatively low methodological quality.

### 2.4. Classification of Modes of Human and Robot Working Together 

In the present analysis, the classification followed by [12,21,50] is incorporated, where five different modes of robots and humans working together may come about:

- Isolation mode, where HRI is never permitted, while normally, barriers are used;
- Coexistence mode, which is similar to the above mode, yet without barriers;
- Synchronization mode, where robot and human focus on different tasks in a synchronized manner and work in different working areas;
- Cooperation mode, where robot and human focus, again, on different tasks, however, working in the same working area;
- Collaboration mode, where robot and human focus on the same task and work in the same working area.

Obviously, the isolation mode refers to conventional robots, commonly used in industry, and together with coexistence mode does not consider any interaction between the human and the robot. In contrast, the other three modes correspond to the gradual increase in the level of human–robot synergy. As stressed in [21], it can be problematic to discriminate the existing mode, as this categorization comes from industry. Furthermore, contemporary user interfaces allow for synergy via virtual shared workspaces. In these cases, the criterion was whether robot and human are working on the same task.

### 2.5. Assessment of the Level of Automation during Decision and Action Stage

**Figure 2:**
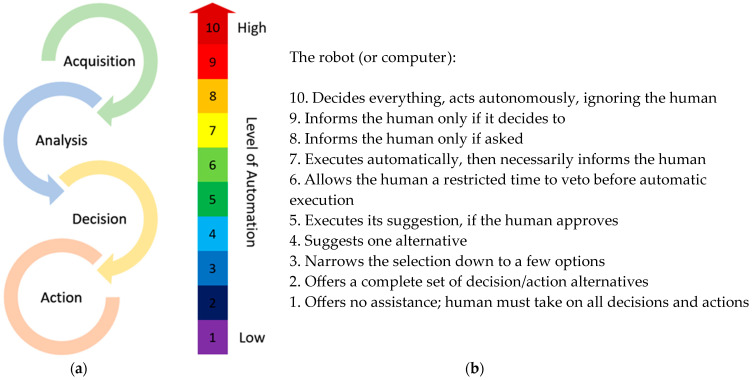
(**a**) A simplified 4-stage model of agricultural applications consisting of information acquisition (acquisition), information analysis (analysis), decision selection (decision), and action implementation (action), and (**b**) The levels of automation for the decision and action stage according to [51].

In general, automation can take place in four stages [51], namely (a) information acquisition (acquisition stage); (b) information analysis (analysis stage); (c) decision selection (decision stage); and (d) action implementation (action stage). Within each of these stages, automation can be realized at a wide range of levels. Following the analysis of Parasuraman et al. [51] for the decision and action stages, a 10-point scale is used in the present study. In this scale, the higher levels characterize increased autonomy of computer (or robot in the present analysis) over human action. Therefore, if a function can be fully carried out exclusively by a human, the lowest level (i.e., “1”) is given, while the higher level (i.e., “10”) denotes that robot decides and acts autonomously. The intermediate levels of automation represent partial automation and different modes of HRI. Indicatively, at level 4, robot proposes an alternative decision, but the human continuously has the authority to either choose another decision/action or prefer the suggested alternative. In contrast, at level 6, the robot gives a limited time for a veto to the human before automatically executing its own decision. The utilized 10-point scale regarding the levels of autonomy, along with the four classes of functions, are shown in Figure 2. In this regard, it should be emphasized that, usually, a range of automation levels are used instead of a unique level, since there may be different alternative situations during HRI [52,53].

## 3. Results

### 3.1. Preliminary Data Visualization Analysis

Data visualization analysis is regarded as an advantageous practical tool to analyze and illustrate massive data amounts, conduct data-driven judgments, interpret the current trends in the research field of interest, and identify research gaps.

#### 3.1.1. Time Distribution

**Figure 3:**
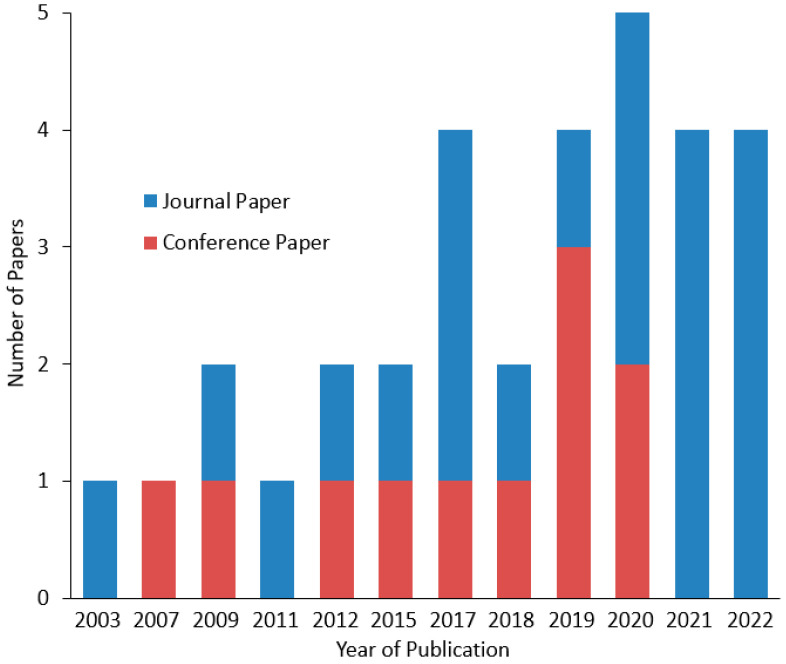
Time distribution of the papers reviewed in the present study.

A preliminary data visualization analysis is presented in this subsection starting from the time distribution of the reviewed studies in Figure 3. As can be deduced from this bar chart, investigation of HRI in agriculture is a recent research field that has concerned scholarly literature for almost the last twenty years, due to the sector-specific extension of “Industry 4.0”. As elaborated in the Introduction, robotics has found fertile ground in agriculture, enlarging their preceding role of performing only non-cognitive and routine missions. However, in contrast with other HRI applications, like those found in industrial settings, rehabilitation and medicine, and education, the peculiar agricultural environment introduces further challenges to the design of synergetic systems. Therefore, only 32 studies were found, most of which were published in recent years. This increase justifies, to some extent, the growing interest in complementary combination of robot and human capabilities in agricultural applications, while also taking advantage of the tremendous progress of ICT.

#### 3.1.2. Distribution of the Contributing International Journals, Conferences, and Disciplines

**Figure 4:**
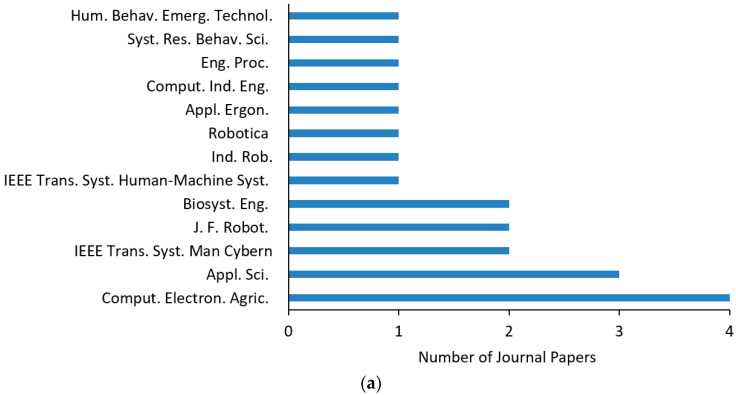

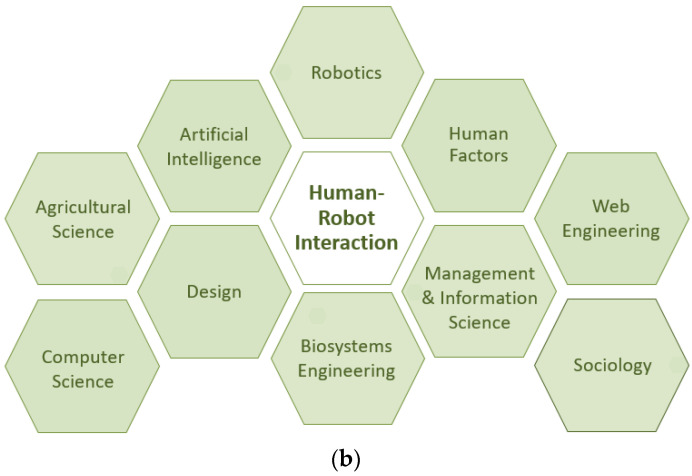
(**a**) Distribution of all contributing international journals and (**b**) different core disciplines engaged in human–robot interaction in agriculture.

Subsequently, the sources where the articles were published were reviewed to determine the research approaches, which drew on knowledge from different disciplines. As can be seen in Figure 4a, “Computers and Electronics in Agriculture” was the main international journal of the current survey. This journal is associated with the application of computer hardware and software to meet the challenges emerging in the framework of smart agriculture, in which robotics is of central importance. Other journals with the same objective, but with less contribution, were “Industrial Robot”, “Journal of Field Robotics”, and “Robotica”, which are not purely interested in the agricultural domain. An interdisciplinary journal with significant contribution was also “Applied Sciences”, which deals with different aspects of applied natural sciences as well as “Biosystems Engineering”. The latter publishes research in engineering for biological systems, including agriculture. “Engineering Proceedings” and “Computers & Industrial Engineering” focus mainly on industrial engineering and the use of computers and electronic communication, which constitute an integral part of it. 

In addition, “IEEE Transactions on Systems, Man, and Cybernetics: Systems” and “Systems Research and Behavioral Science” cover the field of systems engineering with a range of engineering methods, including modeling, simulation, and optimization, and examination of issues from an economic and social perspective. Moreover, a journal aimed at investigating the human factors in the design and management of technical systems at work, namely “Applied Ergonomics”, contributed one article. Finally, “Transactions On Human Machine Systems” and “Human Behavior and Emerging Technologies” include human systems and organizational interactions, system testing and assessment, and cognitive ergonomics in systems and organizations. As far as the selected conference papers are concerned, the biggest contribution was from “IFAC-PapersOnLine” (formerly “IFAC Proceedings Volumes”) and IEEE International Conferences emphasizing robotic systems, including human–robot synergetic systems. In conclusion, several disciplines are engaged in finding innovative HRI solutions in agriculture by redefining problems outside the usual boundaries. Based on the scope and scholarly audience of the above journals and conferences, ten disciplines were identified, which are summarized in Figure 4b, whose theories and methodologies are combined so that unique insights are gained to face the challenges of agricultural environments.

### 3.2. Methodological Quality of the Reviewed Studies

The 32 reviewed papers are summarized in the first column of Table 1 in chronological order: from the first study of Bechar and Edan [52], published in 2003, up to the most recent one of Vásconez and Cheein [54], which was published in November 2022. As mentioned in Section 2.2, the tool developed by Hoy et al. [47] is used in the present study for assessing the methodological quality of the reviewed papers. According to the imposed criteria, all studies proved to be of a high methodological quality (with “++” assigned in the eleventh column), which corresponds to low risk of bias. The items that appeared as more questionable were those related to the quality of the sampling. In some studies, the sampling frame was not a close representation of the target population, since, usually, the authors themselves or a few university students may take part in experimental sessions, sometimes selected in a non-random way. Nevertheless, the implemented methodology was of relatively high quality, counterbalancing this disadvantage.

### 3.3. Brief Review of the Relevant Literature

**Figure 5:**
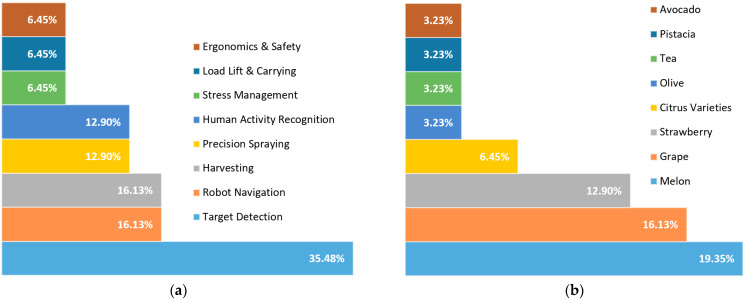

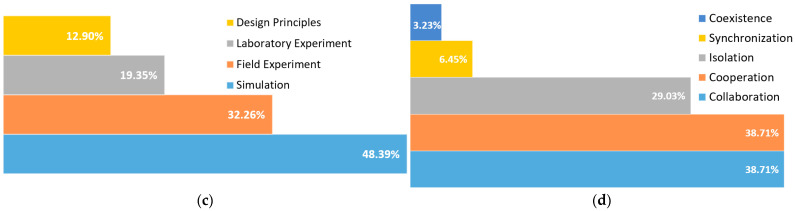
Distribution of the most common aspects investigated in the reviewed studies; (**a**) subject, (**b**) examined crop, (**c**) implemented methodology, and (**d**) interaction mode.

The selected studies are also included in Table 2, whose columns epitomize some important aspects of them, namely the citation of the paper at hand, its subject, the implemented methodology, the examined crop, the interaction mode (based on the taxonomy described in Section 2.4), the automation level (as described in Section 2.5), and the main results. A summary of the aforementioned aspects, which were investigated by the relevant studies, is provided in Figure 5a–d, while a discussion follows immediately after.

Starting from the subject of the reviewed papers (Figure 5a), most of them dealt with a very demanding agricultural task, namely target detection. The key problems come from the peculiar agricultural environment. In essence, occlusion and changing illuminations properties, as well as variability in fruit color, size, shape, texture, orientation, and position, are limiting factors. Apart from the problems related to the location of targets, the uneven and continuously changed terrain and atmospheric conditions make target detection more complicated. Several performance measures have been used for target recognition, including detection time, probability of target detection, and non-target detection (false alarm). Automatic target detection in such environments is characterized by poor performance. Consequently, interaction with humans can be advantageous, considering their superior perception and action capabilities allowing them to adapt to unforeseen agricultural events. 

The majority of the studies associated with implementing HRI for optimizing target detection [52,53,55,56,57,58,59,63] followed a certain methodology for comparing the performance of four different human–robot types of synergy:

- Humans alone detect and mark the targets, while HRI is never permitted. This is compatible with both level 1 in Sheridan’s scale and isolation mode;
- Robots recommend the targets and humans approve and mark them. In particular, the targets are automatically identified with the use of a detection algorithm. Then, humans recognize the algorithm’s true detections by ignoring the false ones and mark the possible missing targets. This interaction corresponds to levels 3–4 in Sheridan’s scale, as mentioned in these studies. In addition, following the analysis described in Section 2.4, this interaction is classified as collaboration, since both robots and humans focus on the same task;
- The targets are automatically detected by the corresponding machine learning algorithm, with the human role being to cancel the false findings, while, like at the above level, the humans marks the missing items. This type of synergy is equivalent to levels 5–7 in the Sheridan scale and, again, is classified as collaboration;
- Purely autonomous marking of targets takes place, in which human intervention is never permitted. Obviously, similar to the first type of synergy that was mentioned above, no HRI exists, demonstrating the highest level of automation in the Sheridan scale, namely 10.

Most of these studies used melons as a target, while grapes were also investigated corresponding in aggregate to approximately 35.5% of all studies (Figure 5b). On all occasions, the collaboration of humans and robots was found to increase detection performance and the corresponding time needed for detection. Both of these outcomes were observed to strongly depend on human decision time. Interestingly, when a field experiment was conducted to evaluate in practice the impact of the synergy on a site-specific spraying application, the proposed collaborative spraying system demonstrated a 50% reduction in the utilized sprayed pesticide [63]. Preliminary laboratory experiments in [82] investigated the opinion of experienced and non- experienced groups on errors produced by machine learning algorithms in a synergistic task.

Moreover, five studies [20,60,62,64,68] investigated robot navigation, which is also a demanding task, because of the particular nature of the rural environment. Adamides et al. [20,62] examined the usability of two types of output devices, two types of input devices, and single or multiple views toward optimizing a teleoperated robotic sprayer, while in [60], a taxonomy was proposed pertaining to usability guidelines. Similarly, Mallas et al. [80] investigated the efficiency of two user interfaces by using two groups in field and simulation experiments, namely computer experts and farmers. Additionally, in [68], the importance of augmented reality was investigated as a means of supervising two autonomous tractors in a test field. Finally, three computational studies [65,67,70] concentrated on greenhouse stress management and how human–robot synergy can both provide higher efficiency and save time.

Focusing on HRI for harvesting applications, Rysz et al. [69,76] developed a risk-averse optimization solution and validated it by using a simulated grove setting, including information for different citrus varieties. A vehicle was successfully implemented in [74] for following the worker during tea plucking, as proved by the experimental field results. Furthermore, Seyyedhasani et al. [71,72] investigated the use of harvest-aid robots for carrying trays to decrease the non-productive walking times of pickers by utilizing data collected from two strawberry fields. In the same vein, to increase situation awareness, in [75,78], wearable sensors were used for gathering data during a human–robot synergistic task involving six sub-activities, which were carried out under different variants. Furthermore, in order to provide more natural means of communication between robot and human, Moysiadis et al. [24] developed a skeleton-based recognition system for hand gestures, which enabled a real-time HRI framework tested in field experiments. In [66], the same robotic system (Thorvald, SAGA Robotics SA, Oslo, Norway) with [24] was used for transporting the picked strawberries, and the opinion of workers on their interaction with it was assessed. For that purpose, a brief questionnaire with a five-point scale was employed.

Aiming at occupational health, which has been recognized as an integral element of collaborative robotic systems, kinetic and kinematic data as well as muscle activation levels were collected in [77] from experienced workers in laboratory experiments to investigate the optimal deposit height of an unmanned ground vehicle. For a similar purpose, Vasconez and Cheein [54] evaluated, in simulated scenarios, the expected production and also the physical workload of workers. Benos et al. [21] examined both ergonomics and safety during HRI operations from an agriculture-oriented perspective, while guidelines for addressing problems in shared environments were described in [61]. Finally, the socio-economic factors driving the shift from pure automation to HRI were analyzed through the prism of a systems thinking approach by Aivazidou and Tsolakis [79]. 

In general, simulated environments were used in the majority of the reviewed studies, while experiments, either in the field or in the laboratory, were also utilized, as well as studies dealing with design principles (Figure 5c). Simulations can be a valuable tool for investigating HRI in agriculture compared to real-world experiments. Benefits associated with simulations are (a) cost-effectiveness, as physical experiments include expensive equipment and land; (b) flexibility to study various scenarios; (c) scalability, enabling researchers to examine large-scale agricultural systems; and (d) risk-free experimentation without the fear of damaging crops or putting human operators at risk. It is worth stressing that simulations cannot fully replicate the intricacies of real-world environments. Therefore, it is essential to validate simulation outcomes by conducting physical experiments. This validation process ensures the dependability and precision of the findings before applying them in real agricultural settings.

Finally, as can be gleaned from Table 2, several automation levels, according to the Sheridan scale, were tested either in field/laboratory experiments or in simulated environments to test the potential of using different interaction levels in agricultural applications. In total, collaboration and cooperation modes, according to the analysis presented in 2.4, were the most usual modes (Figure 5d). 

## 4. Discussion and Conclusions

The present systematic review aimed to shed light on an ever-increasing topic that concerns several sectors worldwide, namely HRI. This emerging research field was methodically analyzed from the perspective of agriculture, which includes complex and dynamic ecosystems as well as live produce highly sensitive to physical and environmental conditions. A comprehensive examination of the present status was carried out by systematically reviewing the relevant literature. In total, 32 scientific papers were found. These studies are a result of the synergistic efforts of multiple disciplines including agricultural sciences, human factors, sociology, and ICT. After an assessment of their methodological approach, the content of the reviewed articles was discussed in terms of their subject, implemented methodology, examined crop, interaction mode, automation level, and main results. 

In summary, most studies dealt with target detection, while studies focusing on detection in combination with precision spraying and/or robot navigation were also observed. Furthermore, simulation was the most preferred methodology, as multiple parameters can be examined. However, field experiments have also been conducted showing encouraging results regarding the benefits of HRI in agriculture. The most studied crops, in descending order of frequency, were melons, grapes, and strawberries, with collaboration and cooperation being the most common interaction modes. These crops have high demands for careful handling, accurate harvesting methods, and precise evaluation of ripeness. Due to the time-consuming and labor-intensive nature of these tasks, the implementation of robotics and automation in these crops can greatly enhance productivity and efficiency. Overall, a range of factors such as the unique attributes of these crops, economic considerations, labor factors, technological feasibility, and research focus collectively contribute to the increased interest in HRI applications, specifically for these high-value crops. These applications can serve as valuable sources of technical knowledge and practices to be disseminated and encouraged among other crop producers. This will aid in the effective adoption of these technologies by considering the requirements, benefits, and potential challenges associated with them. Creating platforms for collaboration and the exchange of knowledge among agricultural growers can bring significant advantages for establishing a supportive ecosystem. 

As can be deduced from the existing literature on HRI in agriculture, the brittleness of autonomous robotic systems in uncontrolled and dynamic conditions in tandem with variability in environments and live produce can result in ineffective operations and production losses. To that end, human workers can complement autonomous systems by overcoming their shortcomings. Nevertheless, the path to fully reap the associated benefits of the capabilities of human–robot synergistic systems is still long. A broad range of research areas is open for further development to meet the needs of reliability and feasibility, thus reaching the stage of being commercially available. As human–robot interactive systems consist of several sub-systems, which should be integrated and coordinated to successfully transfer information and execute tasks as a single unit, several factors should be considered, while various issues must be addressed.

First, considering the tremendous progress in ICT and AI, future research should enable the efficient real-time fusion of a variety of complementary sensors to allow sufficient localization, safe robot navigation, and sensing capabilities. The improvement of coordination issues between humans and robots, through providing robots with a better understanding of human intentions and actions, constitutes a promising research area. Moreover, usability issues pertaining to user interfaces should be tackled. The user interface is the point of interaction between humans and robots, allowing the former to control the robotic system to receive feedback from it and achieve effective operation. Consequently, intensive research efforts are required in the direction of developing user-friendly graphical interfaces (GUI). These interfaces should be able to decrease the mental workload, through methods such as avoiding both the inclusion of software that is not convenient to use and restrictions on the mobility of the operator. Advancement in user interfaces will also enable synergy between humans and teams of light-weight unmanned ground or/and aerial vehicles. This constitutes the next demanding step in HRI for addressing the current challenges and optimizing agricultural practices. Toward that direction, human–robot natural communication frameworks should be improved. With the advancement of big data and the enhanced capabilities of computer hardware, deep learning technology exhibits superior reasoning capabilities compared to traditional machine learning algorithms [83]. Hence, it has gained extensive adoption in industrial domains in recent years, where it has been implemented to solve problems related to communication frameworks, such as hand gesture [84,85] and facial expression recognition under various conditions [32,86]. Likewise, advancement in accuracy of machine learning recognition algorithms can further improve the credibility of wearable-sensor-based multi-posture recognition [87]. 

Future research in the field of HRI in agriculture should give due consideration to the social aspects involved. This entails examining the effects of automation on rural communities, including the exploration of possible changes in skill requirements and socio-economic disparities that may arise [88]. It is imperative to employ user-centered design principles and participatory approaches, actively involving farmers and rural communities during the development process. This approach will ensure that designs are both socially and culturally appropriate, leading to enhanced user acceptance. A deep understanding of social acceptance and trust will be gained by exploring stakeholders’ perceptions and attitudes. Factors contributing to trust building, such as transparency, liability, and accountability, should be taken into account [12,89]. Moreover, ethical considerations, encompassing aspects such as privacy and data security, need to be thoroughly examined. By prioritizing these social aspects in future research, a responsible adoption of robotic systems can be accomplished that aligns with the values and needs of society.

Future research should also put effort into safety aspects in terms of safeguarding workers, crops, and surrounding settings. Also, attention should be paid to the optimal design of HRI systems, including the structure of the team, their specific role, human factors, and complex mechanisms of robotic systems [90,91]. In addition, economic aspects should be investigated in depth regarding the practical use of collaborative robots in agriculture, as farmers will only invest in them on the condition that their investment is going to be profitable after a reasonable time. Future research should also involve the assessment of the environmental implications of using robots, such as their potential to minimize chemical usage and soil erosion and contribute to the advancement of sustainable farming practices. 

The introduction of collaborative robotics is, however, not a trivial issue. It requires open dialogue between stakeholders, clear objectives, proper incentives, and information from policy makers. An effective approach would be the organization of frequent symposiums and workshops that involve farmers in the co-design process. These initiatives can provide a space where farmers, robotics experts, policymakers, and researchers can actively participate in meaningful discussions. By facilitating the exchange of knowledge and experiences, these forums can enable the identification of specific limitations, opportunities, and collaborative solutions. Flexible education and training programs need to be developed to equip agricultural workers with the necessary skills to interact with robotic systems effectively. This can involve tailored training modules on robot usage, maintenance, safety protocols, and troubleshooting. Agricultural extension services, technology providers, and vocational training centers can collaborate to provide tailored hands-on training programs that meet farmers’ specific needs. To study skill competencies compared to emerging robot demands, interdisciplinary research initiatives should also be undertaken focusing on recognizing areas where skills are lacking, assessing how robotics affect job responsibilities, and investigating the social and economic consequences of their implementation. These endeavors may involve cooperation among agricultural scientists, robotics engineers, and behavioral researchers, with the aim to comprehend the human aspects of productive interaction between humans and robots in agricultural environments. 

**Figure 6:**
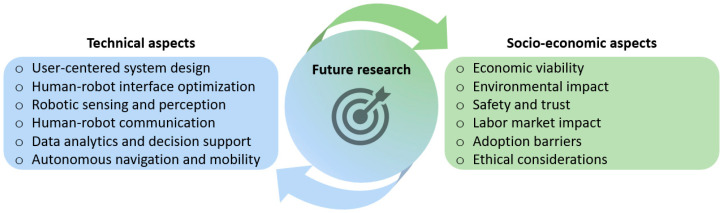
Indicative future research areas that are considered to improve human–robot interaction in agriculture.

The above considerations for future research directions, which were discussed in this section, are summarized in Figure 6.

In conclusion, this review paper presents an extensive evaluation of the present state of HRI in agriculture, emphasizing the progress made, capabilities, technological limitations, and potential applications of this technology within the agricultural domain. Through a comprehensive analysis of the existing literature, this review is expected to serve as a valuable reference for researchers, practitioners, and policymakers who aim to gain insights into the dynamic landscape of agricultural robotics. Finally, by identifying areas necessitating further research and development, this paper seeks to stimulate future innovations and collaborations, thereby fostering the seamless integration of robotics to enhance productivity, sustainability, efficiency, and safety in the agricultural sector.

## Figures and Tables

**Table 1:** Assessment of the methodological quality of the reviewed papers. Note that items 1–4 correspond to external while 5–10 correspond to internal validity [47].

Reference	External Validity				Internal Validity						Overall Quality
	1	2	3	4	5	6	7	8	9	10	11
[52]	Y	N	Y	Y	Y	Y	Y	Y	Y	Y	++
[55]	C	C	C	C	C	Y	Y	Y	Y	Y	++
[56]	C	C	C	C	C	Y	Y	Y	Y	Y	++
[57]	C	C	C	C	C	Y	Y	Y	Y	Y	++
[58]	C	C	C	C	C	Y	Y	Y	Y	Y	++
[53]	C	C	C	C	C	Y	Y	Y	Y	Y	++
[59]	C	C	C	C	C	Y	Y	Y	Y	Y	++
[60]	C	C	C	C	C	Y	Y	C	Y	Y	++
[61]	C	C	C	C	C	Y	Y	Y	Y	Y	++
[20]	Υ	Υ	Υ	Υ	Υ	Υ	Υ	Υ	Υ	Υ	++
[62]	Υ	Υ	Υ	Υ	Υ	Υ	Υ	Υ	Υ	Υ	++
[63]	Υ	Ν	Υ	Υ	Υ	Υ	Υ	Υ	Υ	Υ	++
[64]	C	C	C	C	C	Y	Y	Y	Y	Y	++
[65]	C	C	C	C	C	Y	Y	Y	Y	Y	++
[66]	Υ	Υ	Υ	Υ	Υ	Υ	Υ	Υ	Υ	Υ	++
[67]	C	C	C	C	C	Y	Y	Y	Y	Y	++
[68]	C	Y	C	Y	Y	Y	Y	Y	Y	Y	++
[69]	C	C	C	C	C	Y	Y	Y	Y	Y	++
[70]	C	C	C	C	C	Y	Y	Y	Y	Y	++
[71]	C	C	C	C	C	Y	Y	Y	Y	Y	++
[72]	C	Y	Y	Y	Y	Y	Y	Y	Y	Y	++
[21]	C	C	C	C	C	Y	Y	C	C	Y	++
[73]	C	N	C	Y	Y	Y	Y	Y	Y	Y	++
[74]	N	N	Y	Y	Y	Y	Y	Y	Y	Y	++
[75]	Y	N	Y	Y	Y	Y	Y	Y	Y	Y	++
[76]	C	C	C	C	C	Y	Y	Y	Y	Y	++
[77]	Y	Y	Y	Y	Y	Y	Y	Y	Y	Y	++
[78]	Y	N	C	Y	Y	Y	Y	Y	Y	Y	++
[24]	C	N	C	Y	Y	Y	Y	Y	Y	Y	++
[79]	C	C	C	C	C	Y	Y	Y	Y	Y	++
[80]	C	Y	N	C	Y	Y	Y	Y	Y	Y	++
[54]	C	C	C	C	C	Y	Y	Y	Y	Y	++

“C”: cannot say; “N”: no; “Y”: yes; “++”: high quality (low risk of bias); “+”: acceptable (moderate risk of bias); “−“: low quality (high risk of bias). “1”: Was the study’s target population a close representation of the national population in relation to relevant variables, e.g., age, sex, occupation? “2”: Was the sampling frame a true or close representation of the target population? “3”: Was some form of random selection used to select the sample, or, was a census undertaken? “4”: Was the likelihood of non-response bias minimal? “5”: Were data collected directly from the subjects (as opposed to a proxy)? “6”: Was an acceptable case definition used in the study? “7”: Was the study instrument that measured the parameter of interest shown to have reliability and validity (if necessary)? “8”: Was the same mode of data collection used for all subjects? “9”: Was the length of the shortest prevalence period for the parameter of interest appropriate? “10”: Were the numerator(s) and denominator(s) for the parameter of interest appropriate? “11”: Summary item on the overall risk of bias [47].

**Table 2:** List of the selected papers along with their citation, subject, implemented methodology, examined crop, interaction mode, automation level, and main results.

Ref ^1^	Subject	Method	Crop	Interaction Mode	Automation Level ^2^	Main Results
[52]	Target detection	Lab exp ^3^	Melon	Isolation; Collaboration	1; 3–4; 5–7; 10	Synergy increased the performance by 4% and by 14% compared with the solely manual or autonomous detection, respectively
[55]	Target detection	Simulation	Melon	Isolation; Collaboration	1; 3–4; 5–7; 10	An objective function was developed for evaluating system performance, while the optimal collaboration level may change depending on human and robot sensitivities
[56]	Target detection	Simulation	Melon	Isolation; Collaboration	1; 3–4; 5–7; 10	The best system performance and collaboration level depend on the environment, the task, and the system characteristics
[57]	Target detection	Simulation	Melon	Isolation; Collaboration	1; 3–4; 5–7; 10	Real-time switching of the synergistic levels was accomplished by developed algorithms for increasing system performance
[58]	Target detection	Simulation	Melon	Isolation; Collaboration	1; 3–4; 5–7; 10	Real-time switching of the synergistic levels was achieved, resulting in improved system performance by more than 90%
[53]	Target detection	Simulation	Melon	Isolation; Collaboration	1; 3–4; 5–7; 10	Operational costs were studied, showing that human decision time strongly affects the performance
[59]	Target detection/Precision spraying	Lab exp/Simulation	Grape	Isolation; Collaboration	1; 3–4; 5–7; 10	Four levels of HRI ^7^ were developed and tested, as well as a spraying coverage optimization function
[60]	Robot navigation	Design Principles	N/A	N/A	N/A	A taxonomy was presented and evaluated in terms of an existing user interface for robot teleoperation
[61]	Movements identification	Design Principles	Olive	N/A	N/A	Guidelines are described for addressing problems in sharing human–robot environments
[20]	Robot navigation/Target detection/Precision spraying	Field and lab exp	Grape	Synchronization	1–2	Multiple views, head -mounted display, PC ^4^ keyboard contributed to higher perceived usability
[62]	Robot navigation/Target detection/Precision spraying	Field and lab exp	Grape	Synchronization	1–2	Similar results to [20], while camera placement on the top-back of the robot and on the end-effector improved the surroundings and activity awareness
[63]	Target detection/Precision spraying	Field exp	Grape	Isolation; Collaboration	1; 3–4; 5–7; 10	The collaborative spraying system reduces the sprayed material by half
[64]	Social navigation	Simulation	N/A	Coexistence	N/A	A controller modifies the length of personal space and velocity in order to keep a social distance
[65]	Stress management	Simulation	N/A	Isolation; Cooperation	1–3; 10	Collaboration allows for saving time
[66]	Load lift and carrying	Field exp	Strawberry	Cooperation	N/A	The pilot study showed that the experienced workers positively viewed the cooperation and considered it safe
[67]	Stress management	Simulation	N/A	Cooperation; Collaboration	3–5	The developed protocol provides the highest efficiency as compared to a system without synergy
[68]	Fleet of robots (tele-)operation	Field exp	N/A	Collaboration	3–7	The AR ^5^ system improves the situational awareness of a human for managing a fleet of robots
[69]	Harvesting	Simulation	Orange	N/A	N/A	The developed risk-averse solution minimizes economic costs
[70]	Stress management	Simulation	N/A	Cooperation; Collaboration	3–5	H-R ^6^ synergy can respond to emergency stresses situations fast and effectively
[71]	Harvesting	Simulation	Strawberries and grapes	Cooperation	N/A	Development of model and simulator to predict efficiencies of coupled operations pertaining to manual harvesting and robot transport
[72]	Harvesting	Field exp/Simulation	Strawberry	Cooperation	N/A	Simulations robustness of [71] was evaluated; 5 robots serving as tray-transport from 25 pickers improved efficiency by 10.2%
[21]	Ergonomics and safety	Design Principles	N/A	N/A	N/A	A combined approach is proposed that redefines practical limits, reprioritizes safety measures, and determines the riskiest postures
[73]	Target detection	Lab exp	Strawberry	Collaboration	2–5	Both experienced and non-experienced groups opt for robots producing more false positive results
[74]	Harvesting	Field exp	Tea	Cooperation	N/A	The robot kept on a side-by-side route with two workers
[75]	Human activity recognition	Field exp	N/A	Cooperation	N/A	The prediction of the defined sub-activities demonstrated an 85.6% average accuracy, while fusion of all sensors’ data can yield the maximum accuracy
[76]	Harvesting	Simulation	Citrus varieties	N/A	N/A	H-R collaboration can optimize economic viability of robotic harvesters, especially when it occurs in the early stages of harvesting
[77]	Ergonomics	Lab exp	N/A	Cooperation	N/A	A deposit height of robot equal to 90 cm was suggested by avoiding large lumbar flexion
[78]	Human activity recognition	Field exp	N/A	Cooperation	N/A	Six continuous activities with wearable sensors were performed for a HRI scenario under several variants for obtaining a dataset for ergonomics research
[24]	Human activity recognition	Field exp	Pistacia	Cooperation	5	A real-time skeleton-based recognition framework was developed using 5 hand gestures and successfully tested in field experiments
[79]	Transitioning toward H-R synergy	Design Principles	N/A	N/A	N/A	The interplay among the socio-economic factors and underlying mental models driving the shift from pure automation to HRI are presented via a systems thinking approach
[80]	Robot navigation/Precision spraying	Field exp/Simulation	Grape	Collaboration	1–3	Both groups (computer experts and farmers) made effective use of user interfaces with the tangible one receiving more positive evaluations
[54]	Load lift and carrying	Simulation	Avocado	Cooperation	5	H-R synergy increases the production but necessitates slightly more energy during harvesting

^1^ Ref: Reference; ^2^ automation levels according to Sheridan scale [81]; ^3^ Exp: Experiments; ^4^ PC: Personal Computer; ^5^ AR: Augmented Reality; ^6^ H-R: Human–Robot; ^7^ HRI: Human–Robot Interaction.

## Data Availability

No new data were created or analyzed in this study.
